# 

**Published:** 1818-08

**Authors:** 


In the press, An Enquiry into the Influence of Situation on Pulmonary Consumption, and on the Duration of Life; by John G. Mansford. Illustrated by Statistical Reports.

### CATALOGUE OF MEDICAL BOOKS.

A Supplement to the Pharmacopeias; including not only the Drugs and Compounds which are used by professional or private Practitioners of Medicine, but also those which are sold by Chemists, Druggists, and Herbalists, and for other Purposes. By Samuel Frederick Gray. 8vo. 10s. 6d.

Practical Observations on the Action of Morbid Sympathies, as included in the Pathology of certain Diseases. In a Series of Letters to his Son, on his leaving the University of Edinburgh, in the year 1809. By Andrew Wilson, M.D. 8vo. 9s.

An Essay on the Symptoms, Causes, and Treatment, of Inversio Uteri; with a History of the successful Extirpation of that Organ during the Chronic Stage of the Disease. By W. Mewman, Esq. 8vo. 7s.

Observations on a Stridulous Affection of the Bowels, and on some Varieties of Spinal Disease. With an Appendix of Cases. By J. Bradley, M.D. 8vo. 8s.

The Elements of Forensic, or Juridical Medicine. By George Edward Male, M.D. Second Edition, with Additions. 8vo. 9s.

### *TO CORRESPONDENTS.*

*The following is an extract of a letter from* "A Constant Reader," *who requests information on the subject from our Correspondents.*—"*Seeing in the newspapers that a new Medical Establishment was formed in London, where patients afflicted with Hernias were received and radically cured without surgical operation, I beg the favour, through the medium of your Magazine, of being informed by some Correspondent if such Establishment really exists, and how far so desirable an object as a mode of cure in such cases be actually discovered.—A. C.*"

*The length of Mr. Hutchinson's valuable* "Retrospective View of Chemistry," *necessarily obliges us to defer several Communications until next Month, when their punctual insertion will, we hope, be an apology for their delay.*

*Our Correspondents will please to be particular in the Address of their Communications, which should be thus,*—"To the Editors of the London Medical and Physical Journal, at Mr. Souter's, No. 73, St. Paul's Church Yard; or at Mr. Adlard's, 23, Bartholomew-Close."

